# Proteins Interacting With *Caenorhabditis elegans*  Gα Subunits

**DOI:** 10.1002/cfg.318

**Published:** 2003-10

**Authors:** Edwin Cuppen, Alexander M. van der Linden, Gert Jansen, Ronald H. A. Plasterk

**Affiliations:** 1 Hubrecht Laboratory Uppsalalaan 8 Utrecht 3584 CT The Netherlands; 2 MGC Department of Cell Biology and Genetics and Centre for Biomedical Genetics Erasmus Medical Centre PO Box 1738 Rotterdam 3000 DR The Netherlands

## Abstract

To identify novel components in heterotrimeric G-protein signalling, we performed an extensive screen for proteins interacting with *Caenorhabditis elegans*  Gα subunits. The genome of *C. elegans* contains homologues of each of the four mammalian classes of Gα subunits (Gs, Gi/o, Gq and G12), and 17 other Gα subunits. We tested 19 of the GGα subunits and four constitutively activated Gα subunits in a largescale yeast two-hybrid experiment. This resulted in the identification of 24 clones, representing 11 different proteins that interact with four different Gα subunits. This set includes *C. elegans* orthologues of known interactors of Gα subunits, such as AGS3 (LGN/PINS), CalNuc and Rap1Gap, but also novel proteins, including two members of the nuclear receptor super family and a homologue of human haspin (germ cell-specific kinase). All interactions were found to be unique for a specific Gα subunit but variable for the activation status of the Gα subunit. We used expression pattern and RNA interference analysis of the G-protein interactors in an attempt to substantiate the biological relevance of the observed interactions. Furthermore, by means of a membrane recruitment assay, we found evidence that GPA-7 and the nuclear receptor NHR-22 can interact in the animal.


**Comparative and Functional Genomics**
*Comp Funct Genom* 2003; **4**: 479–491.
Published online in Wiley InterScience (www.interscience.wiley.com). **DOI:** 10.1002/cfg.318

**Primary Research Paper**

### Proteins interacting with *Caenorhabditis elegans* Gα subunits

Edwin Cuppen[1], Alexander M. van der Linden[1], Gert Jansen[2] and Ronald H. A. Plasterk[1]*

[1]*Hubrecht Laboratory, Uppsalalaan 8, 3584 CT, Utrecht, The Netherlands*
[2]*MGC Department of Cell Biology and Genetics and Centre for Biomedical Genetics, Erasmus Medical Centre, PO Box 1738, 3000 DR, Rotterdam, The Netherlands*

**Correspondence to: Ronald H. A. Plasterk, Hubrecht Laboratory, Uppsalalaan 8, 3584 CT, Utrecht, The Netherlands. E-mail: plasterk@niob.knaw.nl*

### Abstract

**To identify novel components in heterotrimeric G-protein signalling, we performed an extensive screen for proteins interacting with *Caenorhabditis elegans* Gα subunits. The genome of *C. elegans* contains homologues of each of the four mammalian classes of Gα subunits (Gs, Gi/o, Gq and G12), and 17 other Gα subunits. We tested 19 of the Gα subunits and four constitutively activated Gα subunits in a large-scale yeast two-hybrid experiment. This resulted in the identification of 24 clones, representing 11 different proteins that interact with four different Gα subunits. This set includes *C. elegans* orthologues of known interactors of Gα subunits, such as AGS3 (LGN/PINS), CalNuc and Rap1Gap, but also novel proteins, including two members of the nuclear receptor super family and a homologue of human haspin (germ cell-specific kinase). All interactions were found to be unique for a specific Gα subunit but variable for the activation status of the Gα subunit. We used expression pattern and RNA interference analysis of the G-protein interactors in an attempt to substantiate the biological relevance of the observed interactions. Furthermore, by means of a membrane recruitment assay, we found evidence that GPA-7 and the nuclear receptor NHR-22 can interact in the animal. Copyright © 2003 John Wiley & Sons, Ltd.**

**Keywords: heterotrimeric G-protein; signal transduction; protein interaction; nuclear receptor**

### Introduction

Heterotrimeric G-proteins, consisting of $\alpha$, $\beta$ and $\gamma$-subunits, are signal transduction molecules that couple ligand-bound seven transmembrane (7-TM) receptors to a wide variety of intracellular second messenger systems (Kaziro *et al.*, 1991; Simon *et al.*, 1991). However, there is a growing body of evidence that 7-TM receptors can also transmit extracellular signals through mechanisms that function independently of G-protein coupling (Brzostowski and Kimmel, 2001). In addition, non-receptor modulators and receptor-independent activators of heterotrimeric G-proteins have been identified (Cismowski *et al.*, 1999; Takesono *et al.*, 1999).

All higher eukaryotic organisms possess G-protein-coupled signal transduction, which has been implicated in many important biological functions, ranging from photoreception to neurotransmission and exocytosis, as well as processes such as embryogenesis, angiogenesis, tissue regeneration, and normal and aberrant cell growth. Analysis of the complete *C. elegans* genome (The *C. elegans* Sequencing Consortium, 1998) resulted in the prediction of 21 Gα, two Gβ, and two Gγ genes (E. Cuppen, unpublished results; Jansen *et al.*, 1999). Besides one member (*gsa-1, goa-1, egl-30* and *gpa-12*) of each of the mammalian Gα classes (Gs, Go/i, Gq and G12), there are 17 *C. elegans* specific genes (*gpa-1–11*, *13–17* and *odr-3*) that most closely resemble the mammalian Go/i

Copyright © 2003 John Wiley & Sons, Ltd.

class but cannot be clearly classified into any of the existing families (Jansen *et al.*, 1999).

The Gs homologue *gsa-1* is required to survive the first larval stage (Korswagen *et al.*, 1997), and *goa-1* (Go/i) and *egl-30* (Gq) reciprocally function in neuromuscular processes that regulate behaviours such as locomotion, egg laying, defecation and pharyngeal pumping (Hajdu-Cronin *et al.*, 1999; Mendel *et al.*, 1995; Miller *et al.*, 1999). Furthermore, *goa-1* and *gpa-16* are functionally redundant in asymmetric cell division (Gotta and Ahringer, 2001). At least 14 of the *C. elegans*-specific G$\alpha$ subunits are thought to function in chemosensory signalling. They are expressed almost exclusively in the specialized structures that sense the environment, such as the amphid neurons, and loss-of-function and over-expression studies revealed chemotactic defects (Jansen *et al.*, 1999; Roayaie *et al.*, 1998). At this point, it is largely unknown how these G-proteins transduce their signal and how the observed specificity in signalling is established.

In this study, we tried to identify new regulatory mechanisms in heterotrimeric G-protein signalling by performing yeast two-hybrid interaction screens. Such screens may result in the identification of both upstream and downstream components in the signal transduction cascade, as well as modulators. We report here on the identification of 11 different interacting proteins, and a systematic approach by which we attempted to establish biological relevance for the observed interactions, using promoter–GFP reporter constructs and loss-of-function studies using RNAi and genetic mutants. Finally, we provide *in vivo* evidence to support the interaction between the *C. elegans*-specific G$\alpha$ subunit GPA-7 and the nuclear receptor NHR-22 using a membrane-recruitment assay.

### Materials and methods

### cDNA cloning

The predicted coding sequence of all *C. elegans* G$\alpha$ subunits was amplified from total RNA (strain N2) by RT–PCR. First strand synthesis was initiated using a mixture of random hexamers and oligo-dT oligonucleotides and cDNAs were amplified by PCR, using specific oligonucleotides that include the start and stop codons and contain recognition sites for restriction enzymes for in-frame cloning in the appropriate two-hybrid bait or prey vector (pGBK-T7 or pGAD-T7, Clontech). Detailed cloning information can be obtained from the authors upon request. The resulting cDNA constructs were checked by sequencing for potential differences with the spliced gene-product predicted by ACeDB and for the absence of mutations. Mutations resulting in constitutive active GTPase-deficient G$\alpha$ subunits were introduced by site-directed mutagenesis using PCR and oligonucleotides harbouring the desired mutation. The amino acid changes that were induced were: GPA-5-QL, Gln203Leu; GPA-6-QL, Gln215Leu; GPA-7-QL, Gln203Leu; GPA-12-QL, Gln205Leu; GOA-1-QL, Gln205Leu; GSA-1-QL, Gln208Leu.

All cDNA sequences were submitted to GenBank: GPA-1, AY008124; GPA-2, AY008125; GPA-3, AY008126; GPA-4, AY008127; GPA-5, AY008128; GPA-6, AY008129; GPA-7, AY008130; GPA-8, AY008131; GPA-10, AY008132; GPA-11, AY008133; GPA-12, AY008134; GPA-13, AY008135; GPA-14, AY008136; GPA-15, AY008137; GPA-16, AY008138; EGL-30, AY008139; GOA-1, AY008140; GSA-1, AY008141; ODR-3, AY008142.

### Two-hybrid library screening and yeast mating assay

Yeast two-hybrid interaction library screenings were done according to the manufacturer's protocol (Clontech). The *C. elegans* prey cDNA library that was screened (mixture of a random primed and a oligo-dT primed mixed stage *C. elegans* N2 cDNA library in pACT) was a kind gift of R. Barstead, OMRF Oklahoma City, OK. For mating assays, bait and prey plasmids were transformed to yeast strain AH109 (*MAT***a**) and Y187 (*MAT*$\alpha$,), respectively, and grown overnight in selective medium lacking tryptophan or leucine, respectively. 10 µl of each culture was mixed in 80 µl YPDA in 96-well microtitre plates in the appropriate matrix and grown overnight at 30 °C with shaking. 5 µl drops were seeded on plates lacking both tryptophan and leucine ($LT^-$) as a control for mating efficiency and on selection plates lacking tryptophan, leucine, histidine and adenine ($LTHA^-$) for detection of interactions.

Copyright © 2003 John Wiley & Sons, Ltd. 

Prey plasmids were rescued from yeast and sequenced. cDNA sequences of the specific interactors were submitted to GenBank: Y45G5AM.1, AF408754; C05D2.6, AF408755; F32A6.4, AF408756; F44A6.1, AF408757; K06A1.4, AF408758; F44B9.6, AF408759; T22A3.3, AF408760; F53A10.2, AF408761; F59H5.1, AF408762; C01H6.9, AF408763.

### Promoter–GFP reporter constructs

To study the *in vivo* expression pattern for candidate interacting genes, we generated PCR fragments harbouring 2–3 kb promoter sequence and the first 1–3 exons of the gene of interest, which are fused in-frame to the GFP coding sequence, as described previously (Hobert *et al.*, 1999). PCRs were performed using Accutaq LA DNA polymerase mix (Sigma, St. Louis, MO), which has proofreading activity. Purified PCR fragments were directly injected at 50 µg/ml in *dpy-20(e1282)* animals together with the plasmid pMH86 (Han and Sternberg, 1991) at 150 µg/ml. For each promoter–GFP reporter construct, we created at least three transgenic lines. Transgenic animals carrying an extra-chromosomal array were analysed under a Zeiss Axioskop 2 fluorescence microscope for GFP expression. Cells were identified in reference to Sulston and Horvitz (1977) and White *et al.* (1986).

### Nematode strains, culturing and manipulation

General methods used for culturing, manipulation, and genetics of *C. elegans* were as described (Lewis and Fleming, 1995). DNA transformation assays in *C. elegans* by microinjection were as described (Mello and Fire, 1995). Deletion mutants were identified as described (Jansen *et al.*, 1997). The following strains were used in this study: Bristol N2, CB1282 [*dpy-20(e1282)*IV], NL561 [*goa-1(pk62)*I], MT363 [*goa-1(n363)*I], DA0823 [*egl-30(ad805)*I], NL795 [*gpa-7(pk610)*IV], NL1586 [*pkIs523(gpa-7-XS)*], NL573 [*pkIs299(hs-goa-1XS)*], CB444 [*unc-52(e444)*II], MT1799 [*lin-36(n766)unc-32(e189)*III], MT555 [*lin-8(n111)-dpy-10(e128)*II], MT111 [*lin-8(n111)*II] and NL4256 [*rrf-3(pk1426)*II]. The CalNuc deletion mutant, *nucb-1(pk1654)*, was identified using oligonucleotides nuc1 (ACACTTCGCGCTCTTT-GTTT), nuc2 (CGTGTTCGATGCAAATATGG), nuc3 (ATGAAAGGCGTACCGATGTC) and nuc4 (ATCGAACATCGGAAAAATCG). A genomic region of about 3.4 kb, including all coding exons for CalNuc (F44A6.1), is deleted in this mutant. In the *nhr-22(n2034)* mutant strain (kindly provided by P. Sengupta and the NemaPharm Group of Axys Pharmaceuticals; Liu *et al.*, 1999) a genomic region of 1.2 kb is deleted (exons 3–5), resulting in an out-of-frame mutation, deleting the ligand binding domain but leaving the DNA-binding domain intact. Male mating efficiency and behaviour of *nhr-22* mutants was assayed as described (Hodgkin, 1983).

### RNA interference

The cDNA inserts of the interacting candidates were subcloned from the prey vector into the *Xho*I site of the L4440 vector (Timmons and Fire, 1998) and transformed into the HT115(DE3) bacterial strain using standard methods. NGM-agar plates containing 1 mM IPTG were inoculated with 50 µl of an overnight bacterial culture and grown overnight. Ten L3-L4 wild-type or *rrf-3* mutant animals (a strain hypersensitive to dsRNA; Simmer *et al.*, 2002) were placed on the plate, incubated overnight and transferred to a fresh plate with nutrient that produces dsRNA of the specific cDNA insert. The $F_1$ progeny was monitored closely for any aberrant behavioural or developmental phenotype.

### Membrane recruitment assay

We made expression constructs driven by the *myo-3* promoter for NHR-22 (pRP2231), NHR-22 fused to GFP (pRP2228), NHR-22 fused to the transmembrane domain of Pat-3 (pRP2232), GPA-7 (pRP2233), GPA-7 fused to GFP (pRP2229), GPA-7 fused to the transmembrane domain of Pat-3 (pRP2234) and GPA-7QL fused to GFP (pRP2230). All transmembrane and GFP tags were fused to the N-terminal end of the proteins. Constructs were injected at 50 µg/ml with 100 µg/ml pMH86 (Han and Sternberg, 1991) into *dpy-20(e1282)* or with 100 µg/ml pRF4 (Kramer *et al.*, 1990) into N2 Bristol animals. At least two transgenic lines were generated for each co-localization experiment. We produced transgenic lines carrying pRP2232 (25 µg/ml) with *gpa-15::gfp* (50 µg/ml) (Jansen *et al.*, 1999) and pMH86 (100 µg/ml) as marker plasmids. Males from these transgenic

Copyright © 2003 John Wiley & Sons, Ltd. 

lines were crossed with transgenic lines carrying pRP2229 or pRP2230 to give rise to transgenic animals that express both *gpa-15::gfp* and *myo-3::gfp::gpa-7WT* (pRP2229) or *myo-3::gfp::gpa-7QL* (pRP2230). GFP expression was observed under a Zeiss Axiskop 2 equipped with a fluorescent light source and Nomarski optics. Laser scanning confocal microscopy was used to visualize GFP expression at the plasma membrane.

### Results

### cDNA cloning of all *C. elegans* Gα subunits

*C. elegans* Gα subunit cDNAs were cloned based upon gene predictions and EST data present in ACeDB (release 12/98). When RT–PCRs failed, predictions were re-examined using three-frame translations of genomic sequence in ACeDB, compared with a multiple protein sequence alignment of the known *C. elegans*, mouse and human Gα subunits. We cloned the cDNAs for all Gα subunits, except for GPA-9, for which we were not able to amplify the 5′ half of the gene, and GPA-17, which was discovered only recently. Sequence analysis shows that about 12% of the intron–exon boundaries (19 out of 154), including the manually corrected start and stop codons, were not correctly predicted by GENEFINDER and annotated in ACeDB (Version WS 12/98). Furthermore, no evidence for alternatively spliced isoforms was found.

### Yeast two-hybrid interaction screen

Each of the cloned *C. elegans* Gα subunits and constitutively active GTPase-deficient mutants of GPA-5, GPA-7, GPA-12 and GSA-1 were used as bait in a yeast two-hybrid interaction screen (Table 1). 815 colonies grew on HTLA- plates, of which 547 turned blue on X-gal. 537 prey plasmids were successfully rescued and used to confirm the interaction specificity in a mating assay with the original and control baits. 100 interactions could not be reproduced and 137 were unspecific. The inserts of the 300 remaining clones were sequenced and compared with database sequences. Based upon the molecular identity, 103 clones were excluded from further analysis, as they encode proteins that reside in cellular compartments such as the mitochondrion, or are extracellular, these being locations in which Gα proteins are not expected to be functional. Furthermore, a set of common false-positives, the heat shock proteins, was discarded (a full list can be obtained from the authors upon request). The remaining 197 candidates were tested in a large yeast mating matrix for their interaction characteristics with all 19 cloned Gα subunits. In this experiment, additional control baits (empty vector, p53, *C. elegans* Gβ subunits) were included. At this point, we found that the original GPA-1 bait contained a frameshift mutation in the middle of the cDNA insert, resulting from a RT–PCR cloning error. We repeated the screen for GPA-1 with a corrected bait and used the frameshifted GPA-1 as a control for unspecific interactions. All other bait inserts were once more sequenced to exclude the presence of mutations and, in addition, all bait inserts were cloned into a bacterial expression vector. No other mutations were found and all bacterially expressed proteins were of the expected size (data not shown).

The majority of the clones (173) was found to interact with one or more of the control baits and thus could be considered unspecific interactors (a full list of the identity of these clones can be obtained from the authors upon request). The remaining 24 candidates did not interact with any of the control baits. As expected, all of these candidates did interact with the bait with which they were originally retrieved from the library. Furthermore, we found that none of the candidates interacted with any of the other *C. elegans* Gα subunits (data not shown), illustrating a high degree of specificity for the identified interactions.

### Characteristics of the interacting candidates

The 24 remaining clones were found to encode 11 different proteins that interact with four different baits (Table 2). To confirm the observed interaction in an independent assay, we switched the bait and prey vector inserts and included GTPase-deficient mutants in a mating assay (Figure 1). Artificial interaction interfaces can be created at the junctions of the DNA binding domains and the bait, and/or the transcription activation domain and the prey. Changing the configuration can eliminate such false-positives, whereas strong interactions are expected to work in both configurations. Four prey inserts activated transcription by themselves, making them uninformative as bait.

Copyright © 2003 John Wiley & Sons, Ltd. 

**Table 1.** Summary of the two-hybrid interaction library screening for all *C. elegans* Gα subunits

| | | Screened | Colonies | X-gal | | Mating | | Sequencing | | Matrix mating | |
|---|---|---|---|---|---|---|---|---|---|---|---|
| | | x $10^6$ | picked | Positive | Negative | Aspecific | Specific | Excluded | OK | Aspecific | Specific* |
| 1 | GPA-1† | 0.6 | 96 | 85 | 15 | 1 | 69 | 42 | 27 | 27 | 0 |
| 2 | GPA-1 | 4.2 | 65 | 59 | 9 | 20 | 30 | 2 | 28 | 28 | 0 |
| 3 | GPA-2 | 1 | 35 | 10 | 7 | 3 | 0 | | | | |
| 4 | GPA-3 | 1.1 | 55 | 33 | 3 | 2 | 28 | 21 | 7 | 7 | 0 |
| 5 | GPA-4 | 1.3 | 30 | 11 | 9 | 0 | 2 | 0 | 2 | 2 | 0 |
| 6 | GPA-5 | 0.5 | 2 | 0 | | | | | | | |
| 7 | GPA-6 | 1.3 | 53 | 42 | 10 | 9 | 23 | 2 | 21 | 15 | 6 (1) |
| 8 | GPA-7 | 2.1 | 3 | 3 | 1 | 1 | 1 | 0 | 1 | 1 | 0 |
| 9 | GPA-8 | 1.8 | 3 | 1 | 1 | 0 | 0 | | | | |
| | GPA-9‡ | | | | | | | | | | |
| 10 | GPA-10 | 0.9 | 7 | 3 | 1 | 2 | 0 | | | | |
| 11 | GPA-11 | 0.6 | 1 | 0 | | | | | | | |
| 12 | GPA-12 | 0.3 | 3 | 0 | | | | | | | |
| 13 | GPA-13 | 0.5 | 68 | 64 | 4 | 25 | 25 | 5 | 20 | 17 | 3 (2) |
| 14 | GPA-14 | 0.6 | 3 | 2 | 0 | 2 | 0 | | | | |
| 15 | GPA-15 | 3.2 | 1 | 1 | 1 | 0 | 0 | | | | |
| 16 | GPA-16 | 2.9 | 12 | 5 | 2 | 3 | 0 | 0 | 0 | | |
| | GPA-17§ | | | | | | | | | | |
| 17 | ODR-3 | 3.1 | 108 | 73 | 5 | 29 | 39 | 11 | 28 | 28 | 0 |
| 18 | EGL-30 | 4.5 | 86 | 39 | 11 | 1 | 27 | 12 | 15 | 15 | 0 |
| 19 | GSA-1 | 1.8 | 15 | 1 | 0 | 0 | 1 | 0 | 1 | 1 | 0 |
| 20 | GOA-1 | 0.3 | 143 | 108 | 20 | 35 | 53 | 8 | 45 | 31 | 14 (7) |
| 21 | GPA-5-QL | 0.5 | 4 | 0 | | | | | | | |
| 22 | GPA-7-QL | 2 | 15 | 5 | 1 | 3 | 1 | 0 | 1 | 0 | 1 (1) |
| 23 | GPA-12-QL | 1 | 6 | 1 | 0 | 0 | 1 | 0 | 1 | 1 | 0 |
| 24 | GSA-1-QL | 0.9 | 1 | 1 | 0 | 1 | 0 | | | | |
| | Total | 37 | 815 | 547 | 100 | 137 | 300 | 103 | 197 | 173 | 24 (11) |

* Numbers between brackets indicate the number of unique proteins.
† After the two-hybrid screens a frameshift mutation was found in the middle of this bait construct.
‡ We were not able to clone the 5'end of GPA-9 cDNA.
§ This subunit was identified in a recently finished gap in the genome sequence during preparation of this manuscript.

For the other candidates, the interaction was confirmed in the reversed configuration assay, with the exception of T22A3.3 (a protein of unknown function). Interestingly, however, T22A3.3 did interact with K06A1.4 (NHR-22) and was also found to interact with LAG-1 [Su(H)] and MPK-1 (MAP-kinase) in other interaction screens (Walhout *et al.*, 2000), suggesting either broad or unspecific interaction activity. We observed a strong homotypic interaction for Y50F7A.1 (Rap1GAP) and a weak homotypic interaction for F44B9.6 (LIN-36). Interaction analysis with GTPase-deficient mutants revealed that the interactions of the nuclear receptors K06A1.4 and Y45G5AM.1 with GPA-7 and GPA-13, respectively, are independent of the activation state of the Gα subunit. T22A3.3 was found to interact exclusively with the wild-type form of GPA-6 and only two (AGS3.1 and Rap1GAP) out of the seven candidates for GOA-1 interact with the GTPase-deficient mutant of GOA-1.

### Expression analysis of the interacting candidates

Gene-expression patterns for the interacting candidates were obtained by fusing promoter regions to GFP-encoding sequences. Complete and partial overlap in expression pattern for the candidate genes and their respective bait can be observed for a subset of the candidates (Figure 2 and summarized in Table 3). For example, both NHR-22 and GPA-7 are expressed in different types of muscle cells and neurons, but also in the male specific neurons in the tail required for mating (Figure 2I). GOA-1 is ubiquitously expressed in neuronal tissue and in muscles (Mendel *et al.*, 1995; Segalat *et al.*, 1995), and

Copyright © 2003 John Wiley & Sons, Ltd. 

**Table 2.** Properties of proteins interacting with G$\alpha$ subunits

| Protein ID | Description | Bait | *n*[*] | Fragment[†] | Chr | QL[‡] | rev[§] | RNAi[¶] | Mutant | Remarks |
|---|---|---|---|---|---|---|---|---|---|---|
| T22A3.3 | Protein of unknown function | GPA-6 | 6(4) | 121–418 | I | – | – | No obvious phenotype | Not available | Interacts also with LAG-1, MPK-1[‖‖], and K06A1.4; has no clear homologues in other organisms |
| K06A1.4 | Nuclear receptor (NHR-22) | GPA-7-QL[††] | 1 | 271–579 | II | + | nd[‡‡] | No obvious phenotype | *nhr-22:* deletion of ligand binding domain: no obvious phenotype. Also not in *gpa-7* or GPA-7-XS background | DNA-binding domain is not present in the interacting fragment |
| Y45G5AM.1 | Nuclear receptor | GPA-13 | 2(2) | 15–400 | V | + | nd[‡‡] | No obvious phenotype | nd | |
| F15D3.1 | Dystrophin (DYS-1) | GPA-13 | 1 | 1948–2241 | I | + | nd | No obvious phenotype | *dys-1*; hyperactivity and hypercontraction of animal[§§] | |
| C05D2.6 | Protein of unknown function** ) | GOA-1 | 1 | 15–650 | III | – | nd[‡‡] | Sterile progeny or reduced broodsize in *rrf-3* mutant background. | Not available | Has no clear homologues in other organisms |
| C01H6.9 | Contains protein kinase domain | GOA-1 | 1 | 307–920 | I | – | nd | No obvious phenotype; also not in combination with a closely related protein F22H10.5 | Not available | Homology to haploid germ cell-specific nuclear protein kinase (haspin) |
| F44B9.6 | LIN-36 | GOA-1 | 6(5) | 682–962 | III | – | + | No obvious phenotype; also not in *goa-1* or GOA-1 XS background | MuV in combination with LIN8/38/15[¶¶] : *goa-1;lin-8* or *goa-1;lin-36* mutants do not have a synMuv phenotype | Weak homotypic interaction |

| | | | | | | | | | | |
|---|---|---|---|---|---|---|---|---|---|---|
| F59H5.1 | Protein of unknown function | GOA-1 | 1 | 501–742 | ‖ | – | + | No obvious phenotype; also not in *goa-1* or GOA-1 XS background | Not available | Has no clear homologues in other organisms |
| F32A6.4‖ | AGS3.1, LGN, Pins (partner of inscuteable) | GOA-1 | 2(2) | 432–579 | X | + | + | No obvious phenotype; also not in *goa-1* or GOA-1 XS background | Not available | Interacting fragment contains three GoLoco motifs out of four |
| F53A10.2‖ | Rap1GAP | GOA-1 | 2(2) | 2–694 | ‖ | + | + | No obvious phenotype; also not in *goa-1* or GOA-1 XS background | Not available | Interacting fragment contains 1 GoLoco motif; homotypic interaction |
| F44A6.1‖ | Nucleobindin/CalNuc EF-hand Ca-binding protein | GOA-1 | 1 | 1–453 | X | – | nd‡‡ | No obvious phenotype, also not in *goa-1* or GOA-1 XS background | *nucb-1*: complete coding region deleted: no phenotype (also not in *goa-1* or *egl-30* background) | No homologues in *C. elegans*, one orthologue in *Drosophila*, two orthologues in mouse and human |

* Number of clones identified in the screen. Between brackets the number of independent clones is indicated.
† Smallest region of the protein sufficient for interaction.
‡ Interaction with GTPase-deficient form of the original bait.
§ Interaction detectable when bait and prey protein are switched.
¶ Results are in line with data published by Fraser *et al.* (2000), Gonczy *et al.* (2000) and Miyabayashi *et al.* (1999).
‖ Interologs; interaction between homologous proteins was previously described in another species
** previously annotated as the homologue of human LIS-1 protein; now the gene is split in two and the interacting fragment has no homology to any known protein.
†† Does also interact with wild-type GPA-7.
‡‡ nd, not determined, because these baits autoactivate the reporter genes.
§§ Bessou, *et al.* (1998);
¶¶ Thomas and Horvitz (1999);
‖‖ Walhout *et al.* (2000).

Copyright © 2003 John Wiley & Sons, Ltd.

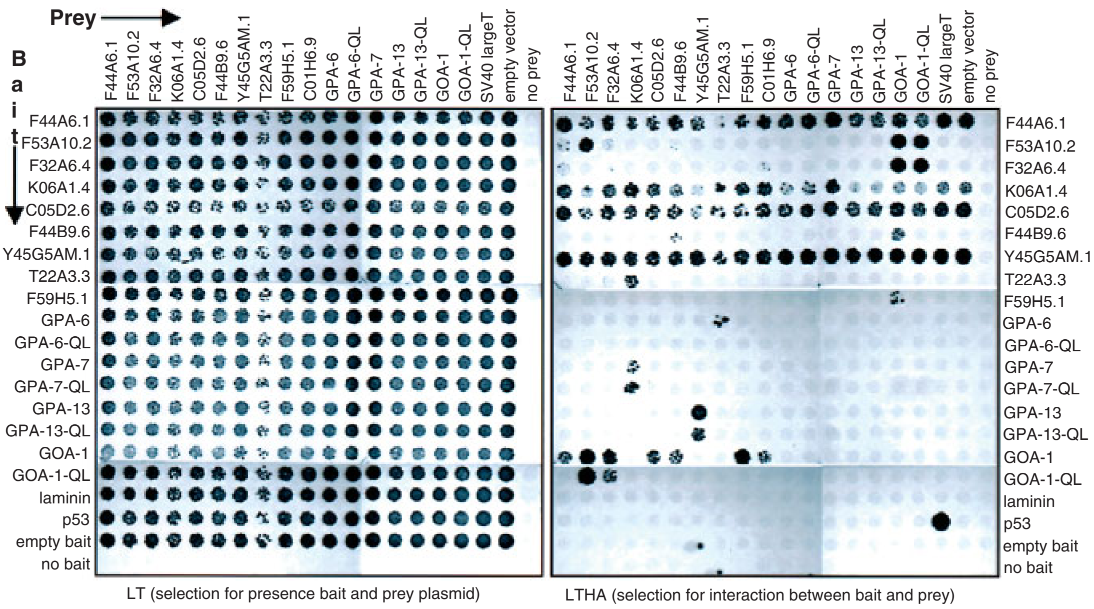

**Figure 1.** Yeast mating assay. The interacting candidates identified in the screen and the corresponding G$\alpha$-subunits were cloned in both bait and prey vectors and tested in a mating matrix. Diploid strains were plated on Leu$^-$/Trp$^-$ medium (left panel) to control for the presence of both bait and prey plasmids, and on Leu$^-$/Trp$^-$/His$^-$/Ade$^-$ medium to select for two-hybrid interactions. Baits F44A6.1, K06A1.4, C05D2.6 and Y45G5AM.1 are not informative, because they autoactivate transcription of the reporter genes several of the interacting candidates are predominantly expressed in muscle cells such as AGS3.1 (Figure 2C–E) and CalNuc, or neuronal cells like LIN-36, F59H5.1 and C01H6.9, whereas others are expressed in both neurons and muscle cells such as C05D2.6.

As for human haspin, expression of the *C. elegans* haspin homologue C01H6.9 is mainly observed in the nucleus (Figure 2G). Some candidates seem to have no or little overlap with their respective bait. T22A3.3 is expressed in several neuronal cells, including a specific chemosensory neuron, AWB (Figure 2H), but its binding partner GPA-6 is expressed in the chemosensory neurons AWA, ASI and PHB (Jansen *et al*., 1999).

We cannot exclude the possibility that the observed expression patterns are not complete, due to the lack of important regulatory promoter elements or to low expression levels. Furthermore, we may have missed co-expression in earlier developmental stages, as we determined the specific cells in L4 and adult stages. However, our data does show a correlation between the expression of the G$\alpha$ subunits and their specific interacting partners.

### Functional analysis of the G$\alpha$ interactors

We performed RNAi experiments (Timmons and Fire, 1998) to functionally inactivate the genes encoding the proteins interacting with G$\alpha$. Wild-type (N2) and a mutant strain (*rrf-3*) that is hypersensitive to dsRNA (Simmer *et al*., 2002) were fed bacteria expressing dsRNA derived from the cDNA inserts of the prey clones. $F_1$ progeny was analysed for aberrant behavioural or developmental phenotypes (e.g. lethality, brood size, development, locomotion and egg-laying activity, morphology, etc.). Only RNAi of C05D2.6 in an *rrf-3* mutant background resulted in a phenotype, e.g. sterile progeny (44%) and a reduced brood size (13 $\pm$4, $n$ = 27). In a wild-type background, no obvious phenotype for C05D2.6 RNAi is observed (brood size is 223 $\pm$21, $n$ = 23). The C05D2.6 (RNAi) phenotype correlates with the reduced broodsize observed in *goa-1* genetic mutants (Mendel *et al*., 1995) and animals fed with dsRNA directed against GOA-1 (our own observations; Fraser *et al*., 2000). Interestingly, both GOA-1 and C05D2.6 are co-expressed in the distal tip cell of the gonad (Figure 2A; Mendel *et al*., 1995), and ablation of this cell is

Copyright © 2003 John Wiley & Sons, Ltd. 

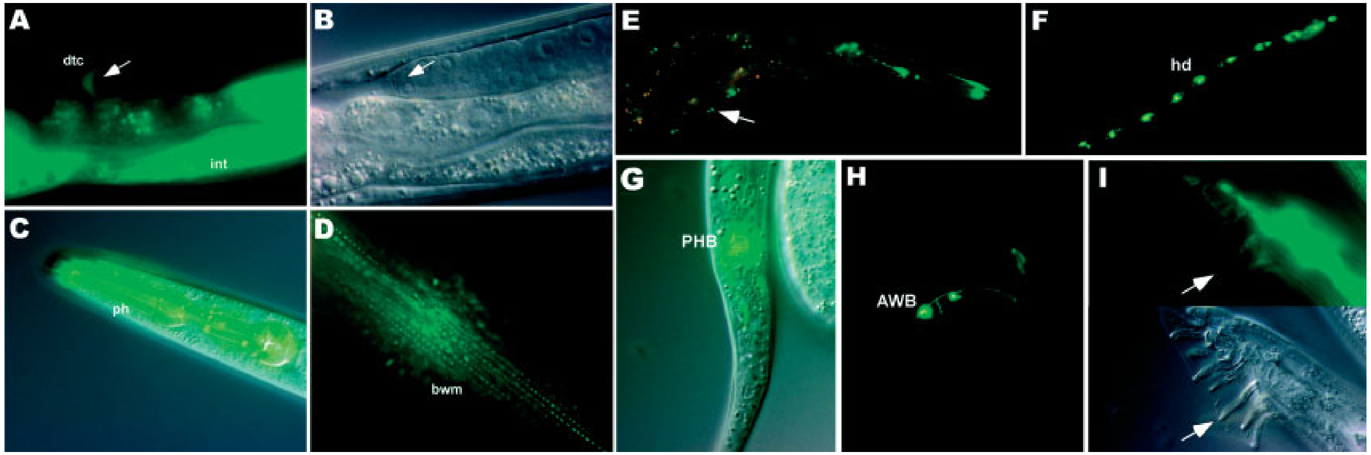

**Figure 2.** Expression pattern of C05D2.6 (A–B), AGS-3.1 (LGN/Pins; C–E), Rap1GAP (F), C01H6.9 (haspin; G), T22A3.3 (H) and NHR-22 (KO6A4.1; I), as determined by promoter–GFP reporter constructs. An overview of all expression patterns is shown in Table 3. (A) C05D2.6 is expressed in the distal tip cell (dtc, arrow) of the gonad and in the intestine (int). (C) Expression of AGS-3.1 is seen in the pharynx (ph), (D) in body wall muscles (bwm) and (E) in a punctuated pattern in the ventral nerve cord (arrow). (F) Expression of Rap1GAP in hypodermal cells (hd). (G) C01H6.9 expression is shown in one of the phasmid neurons, PHB. (H) Expression of T22A3.3 is seen in a set of neurons in the head, including AWB. (I) K06A4.1 expression is seen in male-specific neurons (arrow)

Copyright © 2003 John Wiley & Sons, Ltd.

**Table 3.** Expression profile of the interacting candidates and their corresponding Gα subunits

| | Description | Bait | Expression |
|---|---|---|---|
| T22A3.3 | Unknown | GPA-6 | Neurons in the head (AWB, ADF, ASG (very faint), various interlabial sensory neurons), socket cell, sheath cell in tip of nose, pharyngeal muscle (anterior strong, posterior weak), intestine |
| K06A1.4* | NHR-22 | GPA-7-QL | Neurons in the head (including sensory neurons and various interlabial neurons), interneuron (PVT), many neurons in male tail, pharyngeal and vulval muscle, coelomyciten, intestine |
| Y45G5AM.1 | Nuclear receptor | GPA-13 | Intestine, weak in hypodermal cells, predominantly nuclear staining |
| F15D3.1† | DYS-1 | GPA-13 | Muscles |
| C01H6.9 | Kinase | GOA-1 | Sensory neurons (PHB) and interneurons (PVT) in tail, several uncharacterized neuronal cells in the head with projection to the nose and into the nerve ring, intestinal cells, weak expression in hypodermal seam cells, mainly nuclear staining |
| C05D2.6 | Unknown | GOA-1 | Several neurons in head and tail, pharyngeal muscle, intestine, distal tip cell of the gonad, weak expression in the ventral nerve cord |
| F32A6.4 | AGS3.1 | GOA-1 | All muscles (body wall, pharyngeal, sphincter, vulval), intestine, weak expression in a set of neurons in the head and neurons in the ventral nerve cord, typical subcellular localization in strong dots |
| F44A6.1 | CalNuc | GOA-1 | Pharyngeal muscle, body wall muscle, vulval muscle, weak expression in neurons in head and tail, weak staining in intestinal cells (mainly posterior) |
| F44B9.6‡ | LIN-36 | GOA-1 | Vulval (precursor) cells, many neurons in head, tail and ventral nerve cord; weak expression in hypodermal and intestinal cells; nuclear localization |
| F59H5.1 | Unknown | GOA-1 | Neuronal head ganglia, intestinal cells, hypodermal cells, coelomyciten, highly expressed in embyros |
| F53A10.2 | rap1GAP | GOA-1 | Hypodermal seam cells, various neurons, intestinal cells |
| GPA-6§ | | | Subset of sensory neurons (AWA, ASI (faint), PHB) |
| GPA-7§ | | | Many neurons, muscle cells, many neurons in male tail |
| GPA-13§ | | | Subset of sensory neurons (ADF, ASH, AWC, PHA, PHB) |
| GOA-1¶ | | | Ubiquitously in neurons and muscles, distal tip cell of the gonad |

* A similar expression pattern was described in Miyabayashi *et al*., (1999).
† Bessou *et al*. (1998);
‡ Thomas and Horvitz (1999);
§ Jansen, *et al*. (1999);
¶ Mendel *et al*. (1995).

shown to result in a decreased number of germ cells (Kimble and White, 1981).

Furthermore, RNAi against the candidate proteins that interact with GOA-1 in a mutant background did not modulate the *goa-1* loss- and gain-of-function phenotypes (Table 2). As knockdowns by RNAi may not be complete loss-of-function, we analysed three genetic mutants of interacting candidates: *nhr-22* (K06A4.1), *lin-36* (F44B9.6; Thomas and Horvitz, 1999) and *nucb-1* (F44A6.1) but did not observe any phenotype for these mutants. As *lin-36* has a synthetic multivulva phenotype when combined with mutations in class A synMuv genes (e.g. *lin-8*), we generated double mutants between *goa-1* and *lin-36* or *lin-8*. However, no synMuv phenotype was observed. Although there is only a single homologue for *nucb-1* in *C. elegans*, whereas there are two in mammals, mutants behave completely like wild-type animals and no modulation of *goa-1* loss- or gain-of-function mutant phenotypes could be observed (Table 2), suggesting a non-essential role for this protein in *C. elegans*. Similarly, for a genetic mutant of *nhr-22,* no behavioural, developmental or male-mating defects were observed, nor modulation of *gpa-7* loss- or gain-of-function mutant phenotypes (Table 2). As NHR-22 is a member of a very large, well-conserved gene family in *C. elegans* of more than 250 members (Enmark and Gustafsson, 2000; Miyabayashi *et al*., 1999; Sluder and Maina, 2001), functional redundancy, similar to that observed for the *C. elegans* Gα protein family (Jansen *et al*., 1999), cannot be excluded.

Copyright © 2003 John Wiley & Sons, Ltd. 

### GPA-7 and NHR-22 interact *in vivo*

Because *nhr-22* mutant animals do not show any obvious phenotypes, we examined whether there is *in vivo* evidence to support an interaction using a membrane recruitment assay. We co-expressed GFP coupled to wild-type or activated GPA-7 and NHR-22 coupled to a membrane localization signal in muscle cells. In the absence of NHR-22 (not shown) or the presence of wild-type NHR-22 (Figure 3A), wild-type GPA-7 is localized in both the nucleus and cytoplasm. However, in the presence of membrane-targeted NHR-22, wild-type GPA-7 is recruited to the plasma membrane (Figure 3B). Similar data was found for activated GPA-7 (not shown), consistent with the two-hybrid data (Figure 1). Furthermore, the reversed experiment with GFP-tagged NHR-22 (which is, like GPA-7, localized in the cytoplasm and the nucleus) co-expressed with membrane-targeted GPA-7, results in the same membrane recruitment (not shown). Taken together, these results show that GPA-7 and NHR-22 can interact both *in vitro* and *in vivo*. Such an interaction between a G-protein and a nuclear receptor has not (to our knowledge) been reported before.

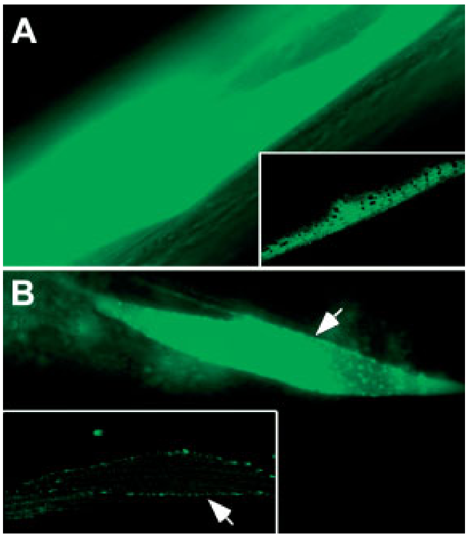

**Figure 3.** *In vivo* interaction between GPA-7 and NHR-22. GFP-tagged GPA-7 is localized in the cytosol and nucleus of muscle cells in the presence of untagged NHR-22 (A) and is recruited to the membrane in the presence of NHR-22 tagged with a membrane localization signal (B), as indicated by multiple foci (arrow). The panels inside the pictures show a confocal image of the muscle cell

### Discussion

This study aimed to identify specific components in G protein signalling pathways by systematically using all *C. elegans* Gα subunits in yeast two-hybrid interaction screens. Although initially a large set of potential interactors was obtained, application of a series of controls resulted in a specific, but surprisingly small, set of only 11 different *C. elegans* proteins that interact with four Gα subunits. Among these are orthologues of proteins known to interact with Gα subunits and novel proteins with homologues in mammalian species.

The low number of interactors identified raises the questions: why did we not identify more interacting proteins?; and why did we not find interactions for the majority of the Gα subunits? First, the Gα or partner protein may not be folded properly, due to its fusion to the DNA-binding or transcription activation domain, prohibiting an interaction. Second, the proximity of the plasma membrane or the presence of additional co-factors may be necessary for proper protein folding or interaction. Indeed, we failed to reconstitute a trimeric complex of an $\alpha$-, $\beta$- and $\gamma$-subunit in a yeast three-hybrid system (E. Cuppen, unpublished results; data not shown), although we were able to demonstrate two-hybrid interactions between $\beta$-, $\gamma$- and $\gamma$-like-subunits (van der Linden *et al.*, 2001). Thirdly, Gα subunits may have to be in their active, GTP-bound, state to interact with effectors. However, we only identified a single protein (K06A1.4) in the four screens with the GTPase-deficient mutants and none of the expected downstream effectors, such as adenylyl cyclases (Gilman, 1987) or phospholipases (Simon *et al.*, 1991), were retrieved. The latter may be due to the way Gα proteins activate these downstream effectors. When these are short-lived 'kiss-and-goodbye'-type interactions, it may be impossible to detect them in an *in vivo* environment, limiting this screen to the identification of more structural interactions. Finally, it is possible that the interacting candidates are not present in the library that was screened, due to under-representation as a result of low expression levels, as is the case for some of the Gα subunits, which are expressed in only a few specific neuronal cells (Jansen *et al.*, 1999). Illustrative of this is that most of the GPA sequences (except for GPA-4, GPA-12, GOA-1, GSA-1, EGL-30) are not present in EST and/or cDNA libraries (source: Wormbase).

Copyright © 2003 John Wiley & Sons, Ltd. 

For the reasons mentioned, the two-hybrid interaction system chosen will not result in the identification of all up- and downstream interactors for Gα proteins. However, we identified both known interactors (Rap1GAP, CalNuc and AGS3.1), thus validating our screen, and novel interacting proteins including the nuclear receptors NHR-22 and Y45G5AM.1 and the homologue of human haspin, C01H6.9, illustrating the value of the approach.

What is the biological relevance of the observed interactions? First, using GFP–reporter constructs, we showed that there is a correlation between the expression in specific cells or cell types of the interacting proteins and their partner. Of the 11 preys, three had a complete overlap (C05D2.6, AGS3.1, and CalNuc) and four had a partial overlap (NHR-22, C01H6.9, LIN-36, and rap1GAP), but for four no overlap was found (T22A3.3, Y45G5AM.1, DYS-1 and F59H5.1). Differences in expression may indicate that the interaction should be considered false-positive, but may also reflect tissue- or cell-specific functions in the case of partial overlap, or be a result of the technical limitations of the approach (GFP sensitivity and silencing of transgenes in early embryonal stages).

Second, we performed RNAi experiments to inactivate gene function. However, except for C05D2.6 in an *rrf-3* mutant background, we did not observe any obvious phenotype using this approach. This may be caused by the predominant neuronal localization of both Gα subunits and interacting partners, where RNAi by feeding is known to be less effective (Timmons *et al*., 2001). Moreover, chromosome-wide analyses have shown that RNAi mimics the genetic knock-out phenotype in only about 50% of cases (Fraser *et al*., 2000; Gonczy *et al*., 2000), making results obtained by this method not fully conclusive. Nevertheless, the lack of phenotype in both RNAi experiments and when using genetic mutants, as shown for three of the interactors, may be associated with a non-essential or redundant role for these proteins. Particularly for NHR-22, redundancy may be an issue, as the nuclear receptor family, together with the G protein-coupled receptors, belongs to one of the largest in *C. elegans*. Interestingly, as for the Gα subunits, many of the nuclear receptors are expressed in (a limited set of) neuronal cells. Nevertheless, we do provide support for an *in vivo* interaction between NHR-22 and GPA-7 using a membrane recruitment assay. As this assay does not formally prove that the endogenous proteins interact *in vivo*, other assays such as co-precipitation or co-localization assays for endogenous proteins will be needed. However, these approaches would require highly specific antibodies for the individual members of these large gene families of nuclear receptors and Gα-proteins, which might be hard to obtain.

While large-scale two-hybrid screens have been shown to be helpful for uncovering networks of interactions in biological processes (Drees *et al*., 2001; Walhout *et al*., 2000), our systematic study of the individual members of the complete Gα-protein family has resulted in only limited results. Although the combination of complete genome information, large-scale two-hybrid screens, and the powerful genetic and molecular techniques that are available for *C. elegans* potentially form an ideal combination for doing (large-scale) functional genomics studies, such an approach may not be suitable for every gene family. In addition, large-scale two-hybrid approaches are now known to be prone to considerable amounts of both false-positives and false-negatives (Mrowka *et al*., 2001; von Mering *et al*., 2002). Therefore, one may have to consider alternative high-throughput technologies, such as mass spectrometry-based approaches like TAP (tandem-affinity purification; Gavin *et al*., 2002) and HMS-PCI (high-throughput mass-spectrometry protein complex identification; Ho *et al*., 2002) to identify relevant interacting proteins and novel candidates in signal transduction cascades.

### Acknowledgements

This work was supported by the Netherlands Organization for Scientific Research (NWO), Grant No. 014-80-008. We thank R. Barstead for kindly providing the cDNA library, P. Sengupta for kindly providing the *nhr-22* mutant strain, and the *Caenorhabditis* Genetics Stock Center for providing some of the strains used in this study.

### References

Bessou C, Giugia JB, Franks CJ, Holden-Dye L, Segalat L. 1998. Mutations in the *Caenorhabditis elegans* dystrophin-like gene dys-1 lead to hyperactivity and suggest a link with cholinergic transmission. *Neurogenetics* **2**: 61–72.

Brzostowski JA, Kimmel AR. 2001. Signalling at zero G: G-protein-independent functions for 7-TM receptors. *Trends Biochem Sci* **26**: 291–297.

Copyright © 2003 John Wiley & Sons, Ltd. 

The *C. elegans* Sequencing Consortium. 1998. Genome sequence of the nematode *C. elegans*: a platform for investigating biology. *Science* **282**: 2012–2018.

Cismowski MJ, Takesono A, Ma C, *et al*. 1999. Genetic screens in yeast to identify mammalian nonreceptor modulators of G-protein signalling. *Nature Biotechnol* **17**: 878–883.

Drees BL, Sundin B, Brazeau E, *et al*. 2001. A protein interaction map for cell polarity development. *J Cell Biol* **154**: 549–571.

Enmark E, Gustafsson JA. 2000. Nematode genome sequence dramatically extends the nuclear receptor superfamily. *Trends Pharmacol Sci* **21**: 85–87.

Fraser AG, Kamath RS, Zipperlen P, *et al*. 2000. Functional genomic analysis of *C. elegans* chromosome I by systematic RNA interference. *Nature* **408**: 325–330.

Gavin AC, Bosche M, Krause R, *et al*. 2002. Functional organization of the yeast proteome by systematic analysis of protein complexes. *Nature* **415**: 141–147.

Gilman AG. 1987. G proteins: transducers of receptor-generated signals. *Ann Rev Biochem* **56**: 615–649.

Gonczy P, Echeverri C, Oegema K, *et al*. 2000. Functional genomic analysis of cell division in *C. elegans* using RNAi of genes on chromosome III. *Nature* **408**: 331–336.

Gotta M, Ahringer J. 2001. Distinct roles for Gα and Gβγ in regulating spindle position and orientation in *Caenorhabditis elegans* embryos. *Nature Cell Biol* **3**: 297–300.

Hajdu-Cronin YM, Chen WJ, Patikoglou G, Koelle MR, Sternberg PW. 1999. Antagonism between G(o)α and G(q)α in *Caenorhabditis elegans*: the RGS protein EAT-16 is necessary for G(o)α signalling and regulates G(q)alpha activity. *Genes Dev* **13**: 1780–1793.

Han M, Sternberg PW. 1991. Analysis of dominant-negative mutations of the *Caenorhabditis elegans* let-60 ras gene. *Genes Dev* **5**: 2188–2198.

Ho Y, Gruhler A, Heilbut A, *et al*. 2002. Systematic identification of protein complexes in *Saccharomyces cerevisiae* by mass spectrometry. *Nature* **415**: 180–183.

Hobert O, Tessmar K, Ruvkun G. 1999. The *Caenorhabditis elegans* lim-6 LIM homeobox gene regulates neurite outgrowth and function of particular GABAergic neurons. *Development* **126**: 1547–1562.

Hodgkin J. 1983. Male phenotypes and mating efficiency in *Caenorhabditis elegans*. *Genetics* **103**: 41–64.

Jansen G, Hazendonk E, Thijssen KL, Plasterk RH. 1997. Reverse genetics by chemical mutagenesis in *Caenorhabditis elegans*. *Nature Genet* **17**: 119–121.

Jansen G, Thijssen KL, Werner P, *et al*. 1999. The complete family of genes encoding G proteins of *Caenorhabditis elegans*. *Nature Genet* **21**: 414–419.

Kaziro Y, Itoh H, Kozasa T, Nakafuku M, Satoh T. 1991. Structure and function of signal-transducing GTP-binding proteins. *Ann Rev Biochem* **60**: 349–400.

Kimble JE, White JG. 1981. On the control of germ cell development in *Caenorhabditis elegans*. *Dev Biol* **81**: 208–219.

Korswagen HC, Park JH, Ohshima Y, Plasterk RH. 1997. An activating mutation in a *Caenorhabditis elegans* Gs protein induces neural degeneration. *Genes Dev* **11**: 1493–1503.

Kramer JM, French RP, Park EC, Johnson JJ. 1990. The *Caenorhabditis elegans* rol-6 gene, which interacts with the sqt-1 collagen gene to determine organismal morphology, encodes a collagen. *Mol Cell Biol* **10**: 2081–2089.

Lewis JA, Fleming JT. 1995. Basic culture methods. *Methods Cell Biol* **48**: 3–29.

Liu LX, Spoerke JM, Mulligan EL, *et al*. 1999. High-throughput isolation of *Caenorhabditis elegans* deletion mutants. *Genome Res* **9**: 859–867.

Mello C, Fire A. 1995. DNA transformation. *Methods Cell Biol* **48**: 451–482.

Mendel JE, Korswagen HC, Liu KS, *et al*. 1995. Participation of the protein Go in multiple aspects of behaviour in *C. elegans*. *Science* **267**: 1652–1655.

Miller KG, Emerson MD, Rand JB. 1999. Goα and diacylglycerol kinase negatively regulate the Gqα pathway in *C. elegans*. *Neuron* **24**: 323–333.

Miyabayashi T, Palfreyman MT, Sluder AE, Slack F, Sengupta P. 1999. Expression and function of members of a divergent nuclear receptor family in *Caenorhabditis elegans*. *Dev Biol* **215**: 314–331.

Mrowka R, Patzak A, Herzel H. 2001. Is there a bias in proteome research? *Genome Res* **11**: 1971–1973.

Roayaie K, Crump JG, Sagasti A, Bargmann CI. 1998. The Gα protein ODR-3 mediates olfactory and nociceptive function and controls cilium morphogenesis in *C. elegans* olfactory neurons. *Neuron* **20**: 55–67.

Segalat L, Elkes DA, Kaplan JM. 1995. Modulation of serotonin-controlled behaviours by Go in *Caenorhabditis elegans*. *Science* **267**: 1648–1651.

Simmer F, Tijsterman M, Parrish S, *et al*. 2002. Loss of the putative RNA-directed RNA polymerase RRF-3 makes *C. elegans* hypersensitive to RNAi. *Curr Biol* **12**: 1317.

Simon MI, Strathmann MP, Gautam N. 1991. Diversity of G proteins in signal transduction. *Science* **252**: 802–808.

Sluder AE, Maina CV. 2001. Nuclear receptors in nematodes: themes and variations. *Trends Genet* **17**: 206–213.

Sulston JE, Horvitz HR. 1977. Post-embryonic cell lineages of the nematode, *Caenorhabditis elegans*. *Dev Biol* **56**: 110–156.

Takesono A, Cismowski MJ, Ribas C, *et al*. 1999. Receptor-independent activators of heterotrimeric G-protein signalling pathways. *J Biol Chem* **274**: 33 202–33 205.

Thomas JH, Horvitz HR. 1999. The *C. elegans* gene lin-36 acts cell autonomously in the lin-35 Rb pathway. *Development* **126**: 3449–3459.

Timmons L, Court DL, Fire A. 2001. Ingestion of bacterially expressed dsRNAs can produce specific and potent genetic interference in *Caenorhabditis elegans*. *Gene* **263**: 103–112.

Timmons L, Fire A. 1998. Specific interference by ingested dsRNA. *Nature* **395**: 854.

van der Linden AM, Simmer F, Cuppen E, Plasterk RH. 2001. The G-protein β-subunit GPB-2 in *Caenorhabditis elegans* regulates the G(o)α-G(q)α signalling network through interactions with the regulator of G-protein signalling proteins EGL-10 and EAT-16. *Genetics* **158**: 221–235.

von Mering C, Krause R, Snel B, *et al*. 2002. Comparative assessment of large-scale data sets of protein–protein interactions. *Nature* **417**: 399–403.

Walhout AJ, Sordella R, Lu X, *et al*. 2000. Protein interaction mapping in *C. elegans* using proteins involved in vulval development. *Science* **287**: 116–122.

White JG, Southgate E, Thomson N, Brenner S. 1986. The structure of the nervous system of *Caenorhabditis elegans*. *Phil Trans R Soc B Biol Sci* **314**: 1–340.

Copyright © 2003 John Wiley & Sons, Ltd. 
